# Boosting the power performance of multilayer graphene as lithium-ion battery anode via unconventional doping with in-situ formed Fe nanoparticles

**DOI:** 10.1038/srep23585

**Published:** 2016-03-30

**Authors:** Rinaldo Raccichini, Alberto Varzi, Venkata Sai Kiran Chakravadhanula, Christian Kübel, Stefano Passerini

**Affiliations:** 1 Helmholtz Institute Ulm (HIU), Helmholtzstrasse 11, 89081 Ulm, Germany; 2 Karlsruhe Institute of Technology (KIT), P.O. Box 3640, 76021 Karlsruhe, Germany; 3 Institute of Physical Chemistry, University of Muenster, Corrensstrasse 28/30, 48149 Muenster, Germany; 4 Institute of Nanotechnology (INT), Karlsruhe Institute of Technology (KIT), Hermann-von-Helmholtz Platz 1, 76344 Eggenstein-Leopoldshafen, Germany; 5 Karlsruhe Nano Micro Facility (KNMF), Karlsruhe Institute of Technology (KIT), Hermann-von-Helmholtz Platz 1, 76344 Eggenstein-Leopoldshafen, Germany

## Abstract

Graphene is extensively investigated and promoted as a viable replacement for graphite, the state-of-the-art material for lithium-ion battery (LIB) anodes, although no clear evidence is available about improvements in terms of cycling stability, delithiation voltage and volumetric capacity. Here we report the microwave-assisted synthesis of a novel graphene-based material in ionic liquid (i.e., carved multilayer graphene with nested Fe_3_O_4_ nanoparticles), together with its extensive characterization via several physical and chemical techniques. When such a composite material is used as LIB anode, the carved paths traced by the Fe_3_O_4_ nanoparticles, and the unconverted metallic iron formed *in-situ* upon the 1^st^ lithiation, result in enhanced rate capability and, especially at high specific currents (i.e., 5 A g^−1^), remarkable cycling stability (99% of specific capacity retention after 180 cycles), low average delithiation voltage (0.244 V) and a substantially increased volumetric capacity with respect to commercial graphite (58.8 Ah L^−1^ vs. 9.6 Ah L^−1^).

In the ongoing challenge to increase the energy density of lithium-ion batteries (LIBs), graphene^1^ has been intensively investigated as anode materials to replace graphite^2^ since 2008^3^. Most of the reports on the use of graphene as active material^4^ are motivated by the larger lithium uptake with respect to the LiC_6_ stoichiometry for graphite^5^. Early studies^6^,^7^ about lithium storage in single layer graphene claimed, in fact, the achievement of Li_2_C_6_ and Li_3_C_6_ stoichiometries. Unfortunately, as later proved by further investigations^8^,^9^, such phases are unstable upon cycling and the stored lithium being never entirely recovered after the first lithiation. This irreversibility is frequently associated with the use of reduced graphene oxides (RGOs) as LIB anodes^10^,^11^ since these materials^12^ generally show various defects^4^, which enable high specific gravimetric capacity values (e.g., > 2,000 mAh g^−1^)^4^ but poorly reversible adsorption of Li^+^ 13 resulting in considerable capacity losses (i.e., low Coulombic efficiencies), especially in the first cycle. Moreover, the high delithiation potential of RGOs, and the generally low density of such materials, act as stumbling blocks for their use in practical applications^4^,^14^,^15^. As graphene and RGOs, graphite has serious limitations, too. It suffers, for example, from poor lithium-ion storage capability at low temperatures^16^. Furthermore, the specific capacity of graphite rapidly decays when high current loads are applied. In this regard, the development of graphene-based materials has recently emerged as strategy to mitigate some of the issues associated with the use of bare graphene (e.g., layers restacking) while, at the same time, attempting to outperform standard graphite^4^, (e.g., at temperatures < 0 °C)^16^. The addition of different electroactive materials, such as metal and/or metal oxide nanoparticles providing reversible insertion (e.g., TiO_2_), alloying (e.g., Sn) or conversion (e.g., Fe_3_O_4_) reactions with lithium, resulting on higher gravimetric capacities than those of bare graphene and graphite^4^. Indeed, electroactive particles and graphene might enhance each other’s performance, with the former helping to reduce the graphene restacking and the latter providing enhanced conductivity and buffering of eventual volume changes. However, as recently pointed out by Obrovac and Chevrier^15^, investigation about the density and the lithiation/delithiation voltages of such materials are essential parameters which should be provided in order to ensure the progress in the LIB field.

In this respect, we aimed to develop a composite formed by multilayer graphene and Fe_3_O_4_, because of the environmentally-friendliness of the latter compound and its expected ability to increase the rate capability by conversion to metallic iron (i.e., good electronic conductor) upon lithiation^17^. Making use of the ionic liquid microwave-assisted exfoliation method previously developed for graphene production^18^, a novel graphene-based composite^4^ has been synthesized. Noticeably, the composite revealed a carved structure with nested Fe_3_O_4_ nanoparticles, which has been observed and characterized for the first time. A multitude of RGO/Fe_3_O_4_ compounds have been reported in the past years^19^,^20^, all with considerable amounts of iron oxide (usually between 30 and 70 wt.%) aiming to exploit the conversion reaction to increase the specific gravimetric capacity of the anode (causing low energy efficiency and relatively high lithiation/delithiation voltages). Differently, this novel composite, obtained by simultaneous synthesis of multilayer graphene and Fe_3_O_4_ nanoparticles, only contains a minimal amount of iron oxide (i.e., <3 wt. %). However, it possesses a peculiar structure enabling superior power performance. Indeed, when the delithiation voltage is limited to 1 V vs. Li/Li^+^, the carved paths traced by the Fe_3_O_4_ nanoparticles and the unconverted metallic iron formed *in-situ* upon the 1^st^ lithiation, incredibly enhance the anode performance at high currents without affecting the energy efficiency. According to our knowledge, such approach is reported here for the first time.

## Results

### Synthesis summary

**Figure 1:**
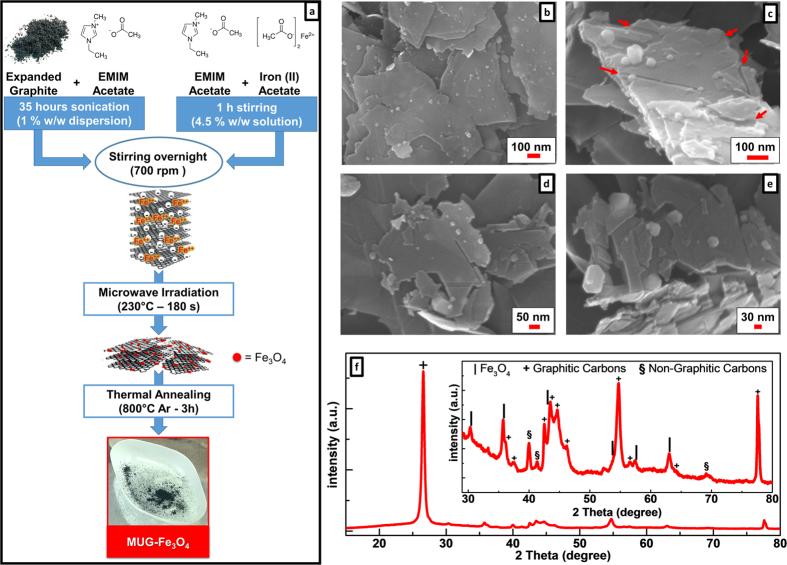
Carved multilayer graphene with nested Fe_3_O_4_ nanoparticles. (**a**) Schematic representation of the synthetic route employed to produce the carved multilayer graphene with nested Fe_3_O_4_ nanoparticles. (**b**,**c**) Low- and (**d**), (**e**) high- magnification SEM micrographs of carved multilayer graphene-Fe_3_O_4_ composite. (**f**) XRD pattern of the carved multilayer graphene-Fe_3_O_4_ composite. Positions of main reflections are marked as (|) Fe_3_O_4_; (+) graphitic carbons; (§) non-graphitic carbons.

The overall procedure for the synthesis of the carved multilayer graphene with nested Fe_3_O_4_ nanoparticles (hereinafter called MUG-Fe_3_O_4_) is illustrated in Fig. 1a. After 35 hours of sonication, the 1% w/w dispersion of expanded graphite (EG) in 1-ethyl-3-methylimidazolium acetate (EMIMAc) is mixed with a 4.5% w/w solution of iron (II) acetate in EMIMAc (previously prepared by 1 h of stirring), and left to stir overnight at 700 rpm. In this first step, the negatively charged thin graphite microplatelets^16^ obtained during the ultrasonication process^21^,^22^, electronically interact with the Fe^2+^ ions. Differently from the widespread procedures employing electrically insulating graphene oxide^10^ as graphene precursor^20^,^23^,^24^,^25^,^26^,^27^, here, by using thin graphite microplatelets, the characteristic π network of the graphitic carbon (and, consequently, the electrical conductive properties of graphite) is preserved. In the following step, the EMIMAc-based dispersion is subjected to microwave irradiation upon which the MUG-Fe_3_O_4_ composite forms. Although rapid and efficient synthesis of metal oxide nanoparticles^28^ with unique structures and properties were already obtained combining ionic liquids and microwaves^29^,^30^, to the best of our knowledge, this is the first time Fe_3_O_4_ is prepared using iron acetate in combination with EMIMAc. It is furthermore worth noticing that, as previously reported and demonstrated^16^,^31^, the microwave heating process reduces the thin graphite microplatelets in size yielding to multilayer graphene (MUG). The composite material was recovered through vacuum filtration and thermally annealed in Ar atmosphere in order to improve its structure and morphology^32^. Finally, the complete removal of residual EMIMAc was performed via thermal decomposition resulting in the formation of benign volatile products.

### Physicochemical Characterizations

As evidenced by the scanning electron microscopy (SEM) micrographs in Fig. 1b, the graphene sheets show jagged edges with irregular facets^33^ along with the presence of nanoparticles. The multilayer morphology of graphene and the nanometric size of the metal oxide particles are revealed in Fig. 1c. Interestingly, a particular morphological feature is observed on the MUG surface where the iron oxide nanoparticles seem to create grooves (e.g., the ones marked by the red arrows) along various MUG edge planes^34^. It is worth noting that a similar structure, to the best of our knowledge, has been reported only twice in literature. These were, however, obtained by means of a completely different synthetic approach (i.e., catalytic hydrogenation of graphite with Nickel nanoparticles^35^,^36^,^37^). At higher magnifications (Fig. 1d,e), the details of the carved structures can be clearly seen and, the nanometric size of the Fe_3_O_4_ particles is better appreciated (10–100 nm range). Apparently, the nanoparticles are able to carve different edge planes of the multilayer graphene causing, especially for the flakes with nanometric lateral sizes, a structural fragmentation on the carbonaceous matrix. When the synthesis is carried out in the absence of graphene (i.e., using only the iron precursor solution), larger Fe_3_O_4_ agglomerates are formed (Supplementary Fig. 1a,b). Moreover, if only the graphite microplatelets dispersion is processed (i.e., without the iron precursor), the obtained multilayer graphene shows a rather different morphology^16^, free of carved structures (Supplementary Fig. 1c,d). The powder diffraction pattern in Fig. 1f allows identifying the different crystallographic features of multilayer graphene and iron oxide. The most intense reflection around 26.5 2 Theta degree is associated with the (002) diffraction plane of graphitic layers^38^. As shown in the inset in Fig. 1f, the other reflections between 35 and 80 2 Theta degree (marked with +), can be ascribable to different graphitic crystalline planes of multilayer graphene, belonging to either hexagonal or rhombohedral phases^38^,^39^,^40^. Additionally, few low-intensity reflections (marked with §) might be due to a limited presence of non-graphitic carbon domains as carbonaceous by-products of the synthetic process^13^,^40^,^41^,^42^,^43^,^44^. Minor reflections corresponding to the Fe_3_O_4_ phase (marked with |) are consistent with the standard XRD data (PDF# 01-073-9877) for the cubic magnetite phase^45^ (Supplementary Fig. 1e). As expected, the powder diffraction pattern of multilayer graphene (Supplementary Fig. 1f) only shows the carbon reflections but no Fe_3_O_4_ signature.

**Figure 2:**
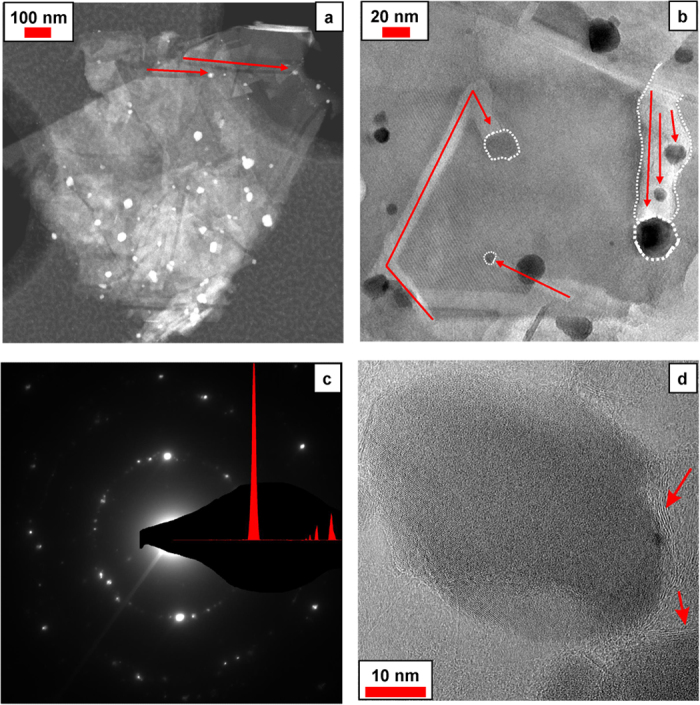
TEM analysis of the composite. (**a**) Scanning transmission electron microscopy (STEM) micrograph of the composite. The carved paths created by the Fe_3_O_4_ nanoparticles are clearly distinguishable (marked with red arrows). (**b**) Bright-field TEM micrograph of the composite where Fe_3_O_4_ nanoparticles are outlined with white round dot dashes and, the carving paths, are indicated with red arrows. (**c**) Corresponding selected area electron diffraction (SAED) pattern for the image in (**b**). (**d**) High resolution TEM (HRTEM) micrograph of a Fe_3_O_4_ nanoparticle covered by a thin graphitic carbon layers originated by the carving of the multilayer graphene (marked with red arrows).

**Figure 3:**
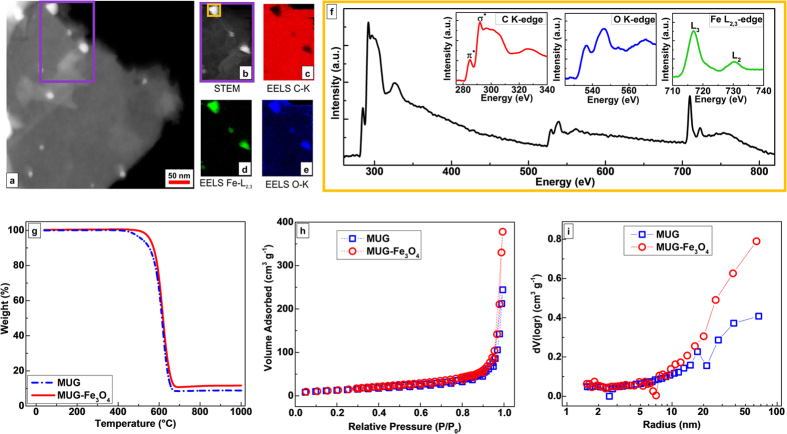
Composite elemental mapping, thermal behaviour and Nitrogen adsorption comparison between multilayer graphene (MUG) and the composite (MUG-Fe_3_O_4_). (**a**) STEM micrograph of a composite region. (**b**) STEM micrograph of the selected area (purple rectangle) in a. (**c**) Carbon elemental mapping of the composite obtained through Electron Energy Loss Spectroscopy (EELS) using the C K-edge. (**d**) Iron elemental mapping of the composite obtained through EELS using the Fe L_2,3_-edge. (**e**) Oxygen elemental mapping of the composite obtained through EELS using the O K-edge. (**f**) EELS spectrum of the selected area (orange rectangle) in b. In the insets, magnified spectrum region, referred to the C K-edge, Fe L_2,3_-edge and O K-edge, are shown. (**g**) TGA profiles (in Oxygen), (**h**) Nitrogen adsorption/desorption isotherms and (**i**) BJH pore size distributions of multilayer graphene and the composite.

Further confirmation for the MUG-Fe_3_O_4_ peculiarity features comes from transmission electron microscopy (TEM) analysis. The scanning TEM (STEM) image shown in Fig. 2a highlights the z-contrast between the MUG graphitic layers and the iron oxide nanoparticles. Here, some of the carved paths traced by the Fe_3_O_4_ nanoparticles (marked with red arrows) can be clearly observed. The bright field TEM micrograph (Fig. 2b) reveals the nanometric size of the iron oxide particles (between 5 and 30 nm in this specific micrograph), which are nested in the carved multilayer graphene matrix. The contrast of the dark Fe_3_O_4_ nanoparticles and the bright grooves (shown by red arrows) generated by the nanoparticles, allow a deeper investigation of the composite structure. Indeed, differently carved paths (width ranging from 10 nm to 40 nm) on different edges planes of the MUG structure can be clearly noticed. In Fig. 2c, the corresponding selective area electron diffraction (SAED) pattern to Fig. 2b is shown. The d-values measured from the SAED pattern fit to the XRD reflections for the iron oxide structure, with lattice spacings of 0.295 nm, while the peaks at ~0.207 nm and ~0.125 nm fit both Fe_3_O_4_ (400) and (622) as well as graphite (100) and (110) reflections. The high resolution TEM micrograph (HRTEM) in Fig. 2d distinctly shows an iron oxide nanoparticle covered by thin graphitic carbon layers (marked with red arrows). According to this micrograph, it is reasonable to assume that MUG fragments, including those generated by the carving, are wrapped around the Fe_3_O_4_ nanoparticles during the composite synthesis. To the best of our knowledge, similar results were only obtained by chemical vapour deposition, as also recently reported by Chang *et al.*^46^ However, EMIMAc can be excluded as source of carbon since it is known that it decomposes exclusively to volatile products upon thermal treatment^47^,^48^ (Supplementary Fig. 2). To further enlighten the MUG-Fe_3_O_4_ structure and composition, additional analyses were performed. The area marked by the purple rectangle in Fig. 3a,b has been investigated by STEM-EELS mapping (looking at the C K-edge, Fe L_2,3_-edge and O K-edge) in order to further confirm the elements present in the composite. In Fig. 3c–e, the elemental maps for C, Fe and O elements are shown, respectively. As expected, C clearly represents the most abundant element, while Fe and O are mostly confined where the nanoparticles are located. Fig. 3f, depicts the EEL spectra of a selected area of the composite (orange rectangle in Fig. 3b). The C K-edge EEL spectrum (see inset in Fig. 3f) shows two features for the near edge fine structure: (i) a narrow peak at around 285 eV, corresponding to the electronic transition from 1 s to π^*^ states^49^, which is characteristic for the carbon sp^2^-hybridization and, (ii) a sequence of broad peaks, ranging from 292 eV to 311 eV, which correspond to transitions from the 1 s to the unoccupied σ*^50^. The shape and the intensity of the latter peaks fits well those of an ordered graphitic structure^51^,^52^, thus supporting the previous XRD analysis. At the same time, the O K-edge and Fe L_2,3_-edge EEL spectra (Fig. 3f inset with blue and green line, respectively) confirm the presence of iron oxide^53^. It is worth noting that no significant amount of N is detected at around 450 eV, thus, confirming the complete ionic liquid removal during synthesis. In Fig. 3g, the TGA curves of both MUG and the composite show the weight loss upon heating. The multilayer graphene is stable, in oxygen, up to 450 °C, however, a loss is observable between 460 °C and 670 °C. At 1000 °C, MUG shows a weight loss of about 91.15%, which is consistent with the results obtained from the CHNS elemental analysis (Table 1)^54^,^55^. The weight loss of MUG is not 100% because of the synthetic process applied. In fact, during the synthesis, thermally stable carbonaceous species (i.e., those denoted as “non-graphitic carbons” in the XRD analysis which also include amorphous carbons) can be obtained, as already reported in literature for graphene and other kinds of carbon^56^,^57^,^58^. Regarding the composite, MUG-Fe_3_O_4_, its stability extends up to 500 °C, i.e., before showing a similar weight loss between 530 °C and 670 °C. Considering the TGA curves of EMIMAc and EG (Supplementary Fig. 2), the 88.32% of MUG-Fe_3_O_4_ weight loss at 1000 °C, is compatible with the presence of the Fe_3_O_4_ in the composite^57^. Indeed, it is possible to calculate the Fe_3_O_4_ amount as 2.73 wt. %, corresponding to a Fe content of about 1.98 wt. %, which is in the same order of magnitude of the ICP result reported in Table 1 (Fe content of about 0.25 wt. %). It is worth pointing out that no Fe was detected by ICP in the EG precursor or MUG (Table 1), thus confirming the presence of iron only in the composite. It should be furthermore noted that the low hydrogen content found in both MUG and MUG-Fe_3_O_4_ is in agreement with the presence of minor non-graphitic carbon domains (as previously revealed by the XRD analysis) and dangling-bond-containing edges^44^. The influence of grooves on the porosimetric features of the composite has been also investigated by Brunauer-Emmett-Teller (BET) surface area and Barrett-Joyner-Halenda (BJH) pore size distribution analyses. From the nitrogen adsorption-desorption isotherms for MUG and MUG-Fe_3_O_4_ (Fig. 3h) similar values of BET surface area are calculated (48.27 m^2^ g^−1^ and 51.79 m^2^ g^−1^ for multilayer graphene and composite, respectively). However, as already reported^16^,^59^,^60^, the graphene sheets’ arrangement play a crucial role in this analysis as it can considerably reduce BET surface area values up to one order of magnitude. According to the BJH analysis (Fig. 3i), the presence of Fe_3_O_4_ has a non-negligible effect on the pores size and volume of the composite. Up to 9–10 nm pore radius, no substantial difference in the pores volume could be noticed. However, above 10 nm the total pore volume increases reaching the final values of 0.79 cm^3^ g^−1^ and 0.41 cm^3^ g^−1^ for MUG-Fe_3_O_4_ and MUG, respectively. The increased pore volume matching the width of the carved path (10–40 nm) confirms the essential role of Fe_3_O_4_ particles in enhancing the porosity of multilayer graphene in the composite material.

### Electrochemical Characterizations

Besides the above-mentioned physicochemical characterizations, the MUG-Fe_3_O_4_ composite was investigated as LIB anode active material. For the sake of comparison, the evaluation of the Li^+^ storage properties of Fe_3_O_4_ synthesized through the same ionic liquid microwave-assisted synthesis was also performed (Supplementary Fig. 3), showing the typical issues of a conversion material (e.g., high voltage hysteresis, capacity fading upon cycling and low coulombic efficiency), which prevent its practical application in LIB^61^.

Regarding MUG, its specific capacity, coulombic efficiency and, in particular, the voltage profiles are similar to those of graphite but, contrary to the latter, it shows acceptable lithium storage capacity even at high specific currents and low temperatures^16^. As all graphenes, MUG generally exhibits low density^4^ and, consequently, its volumetric capacity is smaller than that of graphite. Surprisingly, however, the carbon density in MUG-Fe_3_O_4_ is higher than that in pure MUG, which represents an interesting property.

**Figure 4:**
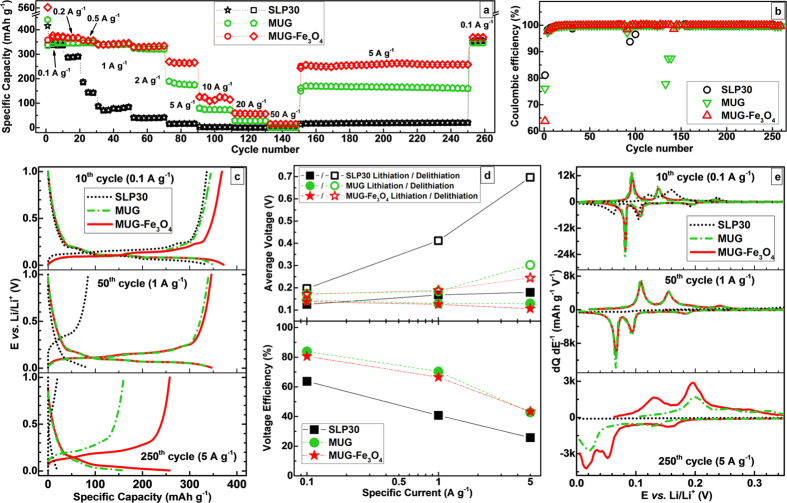
Rate capability test comparison for commercial graphite (SLP30), multilayer graphene (MUG) and composite (MUG-Fe_3_O_4_) in the 0.005 V–1V potential range. (**a**) Specific gravimetric capacities for various galvanostatic lithiation/delithiation cycles performed at different current  rates (measurements performed in Li half-cell with 1 M LiPF_6_ in EC:DMC 1:1 w/w electrolyte) for three different active material, i.e., composite (MUG-Fe_3_O_4_), multilayer graphene (MUG) and graphite (SLP30). (**b**) Coulombic efficiencies for the various galvanostatic lithiation/delithiation cycles reported in a. (**c**) Potential profiles of selected cycles at different specific currents. (**d**) Average lithiation/delithiation voltages and voltage efficiencies for the selected cycles in c. (**e**) Investigation of the lithium ions storage mechanisms: calculated dQ dE^−1^ vs. E differential profiles for the selected cycles in c, in the potential range 0.00 V–0.350 V.

**Figure 5:**
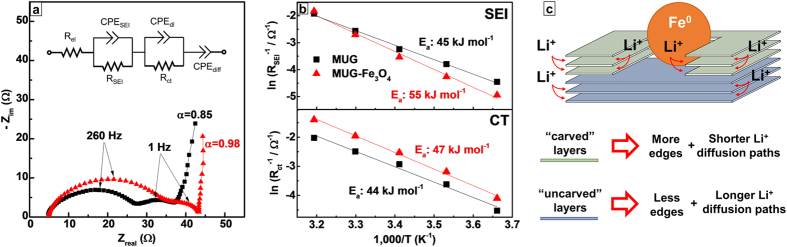
Electrochemical impedance spectroscopy of multilayer graphene (MUG) and the composite (MUG-Fe_3_O_4_). (**a**) Nyquist plots collected after lithiation of the samples up to 0.11 V (measurements performed in a 3-electrodes Li half-cell with 1 M LiPF_6_ in EC:DMC 1:1 w/w electrolyte, at 20°C). (**b**) Arrhenius Plots used to extrapolate the activation energies associated to Li^+^ diffusion through the SEI (upper part), and charge transfer (bottom part). (**c**) Schematic model of the enhanced Li^+^ storage properties provided by the peculiar morphology of MUG-Fe_3_O_4_.

To evaluate the electrochemical performance, MUG-Fe_3_O_4_-based electrodes were investigated in half-cell configuration and subjected to repeated galvanostatic charge/discharge cycles in the narrow voltage range comprised between 0.005 V and 1 V (Fig. 4a). In literature, negative electrodes are frequently delithiated up to 3 V. However, only a restricted operative voltage window is of practical interest, as it results in lower average lithiation/delithiation voltages^15^ (hereinafter ALV and ADV, respectively) and thus higher voltage efficiency (VE) (Supplementary Fig. 4a–c). Of course, limiting the delithiation to 1 V results on the loss of the Fe_3_O_4_ conversion mechanism, which, however, contributes minimally to the composite’s capacity, and only at medium-low currents (Supplementary Fig. 4d). The results in Fig. 4a, especially the comparison with MUG and SLP30 (this latter being a commercial graphite anode material of Imerys Graphite & Carbon), indicate as the metallic Fe nanoparticles, formed during the first lithiation, as well as the carved paths, have a beneficial effect on the high rate performance (i.e., above 2 A g^−1^). During the 1^st^ cycle, MUG-Fe_3_O_4_ obviously showed the highest lithiation capacity due to the conversion reaction of Fe_3_O_4_ to Fe^0^, but, for the same reason, also the lowest CE (i.e., 63.8% vs. 76.1% and 81.1% for MUG-Fe_3_O_4_, MUG and SLP30, respectively, in Fig. 4b) (see the voltage profiles and detailed discussion in Supplementary Fig. 5). After 10 cycles, however, all active materials showed stable and similar performance, with average capacity values of about 335 mAh g^−1^, 348 mAh g^−1^ and 370 mAh g^−1^ for SLP30, MUG and MUG-Fe_3_O_4_, respectively. However, as specific currents higher than 0.2 A g^−1^ were applied, commercial graphite suddenly lost its storage capability. The decay of electrochemical performance is even more dramatic at higher currents, e.g., 1 A g^−1^, where only about 80 mAh g^−1^ were delivered by SLP30. In comparison, under the same current load, both MUG and MUG-Fe_3_O_4_ showed a stable behaviour, providing about 340 mAh g^−1^ and 350 mAh g^−1^ at the 50^th^ cycle, respectively. However, when the applied current was increased to 5 A g^−1^, MUG-Fe_3_O_4_ substantially outperformed MUG, also. Indeed, at the 10^th^ cycle at 5 A g^−1^, an average specific capacity of 15 mAh g^−1^, 180 mAh g^−1^ and 265 mAh g^−1^ were delivered by SLP30, MUG and MUG-Fe_3_O_4_, respectively. Upon prolonged cycling at such high current (i.e., after 180 cycles), MUG-Fe_3_O_4_ offered 258 mAh g^−1^, i.e., about 99% capacity retention respect to the 1^st^ cycle performed at 5 A g^−1^, compared to 165 mAh g^−1^ and 18 mAh g^−1^ of MUG and commercial graphite, respectively. The rather poor performance of the latter material is well explained by the voltage profiles in Fig. 4c, highlighting how the ohmic drop of SLP30 graphite increased dramatically with the applied current. Differently, MUG and MUG-Fe_3_O_4_ showed similar behaviour up to 1 A g^−1^ while, at 5 A g^−1^, MUG-Fe_3_O_4_ achieved considerably higher specific capacity. Fig. 4d (upper part) displays the evolution of ALVs and ADVs, for the three different active material, at different currents. It should be emphasised that ADV, in particular, represents a key parameter for an anode material because it relates with the energy output of the LIB incorporating it^15^. For all the three different active materials, at the lowest current applied (i.e., 0.1 A g^−1^), the ALVs and ADVs ranged from 0.12 V–0.14 V and 0.17 V–0.2 V, respectively. However, when the specific current of 5 A g^−1^ was applied, the ADV of SLP30 rise up to 0.7 V while the ALV is about 0.18 V. On the contrary, MUG and MUG-Fe_3_O_4_, showed ALVs of about 0.13 V and 0.10 V and ADVs of about of 0.30 V and 0.24 V, respectively. For the same selected cycles, the assessment of the voltage efficiency, has been carried out too. VE, which is defined as the ratio of ALV and ADV under specific current conditions^62^, is an excellent tool used to estimate the electrode materials’ impact on the energy efficiency of the battery (for Coulombic efficiency > 99%)^63^. As shown in Fig. 4d (lower part), the VE of SLP30, already small at 0.1 A g^−1^ (i.e., 64%), reach the lowest value of 26% when the highest specific current is applied. Contrarily, both MUG and MUG-Fe_3_O_4_ show similar VEs of about 82%, 68% and 43% for applied current of 0.1 A g^−1^, 1 A g^−1^ and 5 A g^−1^, respectively. To understand the reason behind the lower ALV and ADV of MUG-Fe_3_O_4_ (i.e., its superior ability of storing lithium ions, especially at high currents), differential capacity analysis^64^ has been performed (Fig. 4e). The differential capacity plots of the selected cycles in Fig. 4c show features only in the 0.005 V-0.350 V range, as this is the potential range where the main Li^+^ storage mechanisms occurs. Different lithiation/delithiation regions could be ascribed to different Li-C stoichiometries^65^,^66^, as reported in Supplementary Table 1. Moreover, monitoring the polarization of the electrodes, through peaks’ shifts, the influence of the different Li^+^ storage mechanisms, on the overall electrochemical performance, was identified. With respect to MUG and MUG-Fe_3_O_4_, graphite showed significant polarization associated to the Li^+^ intercalation stages. At 1 A g^−1^ SLP30, in fact, lost almost completely the typical staging behaviour (see also Supplementary Fig. 6a). On the contrary, MUG shows a polarization similar to MUG-Fe_3_O_4_, and all the peaks are still clearly detectable. At the 250^th^ cycle (specific current of 5 A g^−1^), graphite shows only a flat line, thus indicating the complete loss of the staging mechanism (see also Supplementary Fig 6b), while MUG loses the LiC_12_ ↔ LIC_6_ staging process. Contrarily, MUG-Fe_3_O_4_ still enables all lithium intercalation stages, even if they appear largely polarized. This explains the higher capacity of MUG-Fe_3_O_4_ at 5 A g^−1^ which, as a matter of fact, still exhibits the peaks related to stage I. This fact suggests that, the carved paths, together with the presence of metallic Fe nanoparticles, should have a beneficial effect on the kinetics of Li^+^ intercalation into the carved MUG. In order to further understand the influence of both the carved paths and the Fe nanoparticles, electrochemical impedance spectroscopy (EIS) measurements have been performed on MUG-Fe_3_O_4_ and MUG. The Nyquist plots displayed in Fig. 5a highlight a similar response for the two samples. Both materials feature, indeed, two depressed semicircles at high and medium frequencies, which can be associated to the diffusion of Li^+^ in the SEI (R_SEI_ | CPE_SEI_) and charge transfer (R_ct_ | CPE_dl_), respectively. Interestingly, the SEI formed on MUG-Fe_3_O_4_ appears to be more resistive, as also confirmed by the larger activation energy (see Fig. 5b). As reported in previous studies^67^,^68^, the presence of Fe^0^ particles might play a catalytic role for the decomposition of the electrolyte, thus, leading to the growth of a thicker, but more stable SEI. The two samples present comparable activation energies for charge transfer (see Fig. 5b), suggesting that the Fe nanoparticles do not directly affect this process. However, MUG-Fe_3_O_4_ interestingly shows a reduced resistance compared to the MUG. This might be associated to the presence of the grooves in the MUG-Fe_3_O_4_. We indeed propose that, by carving the MUG surface, the nanoparticles create additional graphitic edges where Li^+^ can easily intercalate without affecting the battery performance. This peculiar morphology of MUG-Fe_3_O_4_ would also enable shorter diffusion paths for Li^+^ ions (see Fig. 5c). The low frequency behaviour seems to support this claim. For both samples, the low frequency tail does not show the typical Warburg-like behaviour (α = 0.5), but a much steeper rise, which can be better fitted with a constant phase element (CPE), usually associated to the limit capacitance element^69^. This indicates for a very minor effect of the Li^+^ diffusion in the material solid phase on the overall lithiation/delithiation process. With this regard, the lower slope of MUG (α = 0.85 compared to α = 0.98 for MUG-Fe_3_O_4_) suggests higher hindrance to the solid-state diffusion.

Despite the intriguing mechanism and the highly promising results in terms of Li^+^-ion storage behaviour, we are aware that gravimetric values have only limited relevance when it comes to practical application^15^. Therefore, taking into account the electrode densities (Table 2), specific volumetric capacities were calculated for the three materials (Table 3). The combination of these results with the ADVs allow us to depict a more realistic scenario about their possible use in real LIB applications^15^. At the lowest specific current (i.e., 0.1 A g^−1^), although it exhibits a smaller gravimetric capacity, graphite provides the highest volumetric capacity because of its higher density. At 1 A g^−1^, however, the poor performance of graphite leads to its volumetric capacity being only half that of MUG-Fe_3_O_4_ (40.5 Ah L^−1^ vs. 79.0 Ah L^−1^). Additionally, the ADV dramatically increases for graphite (0.412 V) with respect to MUG and MUG-Fe_3_O_4_, showing rather similar values (i.e., 0.184 V and 0.189 V, respectively). At higher currents (e.g., 5 A g^−1^), MUG-Fe_3_O_4_ outperforms both reference materials, providing a volumetric capacity of 58.8 Ah L^−1^ and an ADV of 0.244 V. On the contrary, graphite only delivered 9.6 Ah L^−1^ with an ADV of 0.697 V. MUG, indeed, showed also a rather low ADV (0.303 V) but, due to its lower density and storage capability than MUG-Fe_3_O_4_, also a limited volumetric capacity (33.7 Ah L^−1^).

## Discussion

Carved multilayer graphene with nested Fe_3_O_4_ nanoparticles has been synthesized through an innovative synthetic pathway. The characteristic structure and morphology of this material has been widely investigated proving its peculiarity in terms of structural arrangement between the Fe_3_O_4_ nanoparticles and the multilayer graphene matrix. Its use as LIB anode revealed enhanced Li^+^ storage properties of this material compared to multilayer graphene and commercial graphite. As evidenced by the differential capacity analysis and EIS measurements, the key role in the advanced battery performance of the composite, is played by: (i) the carved structure of the multilayer graphene and, (ii) the confinement, upon cycling of metallic iron nanoparticles. Indeed, remarkable high values of specific volumetric capacity of 58.8 Ah L^−1^ and low average delithiation voltage of 0.244 V have been achieved upon applied lithiation/delithiation current of 5 A g^−1^.

## Methods

### Synthesis of carved multilayer graphene with nested-Fe_3_O_4_ nanoparticles

The EG-EMIMAc dispersion was prepared by mixing 1.5 g of expanded graphite (EG, SGL Carbon) and 155.5 g of 1-ethyl-3-methylimidazolium acetate (EMIMAc, Ionic Liquids Technologies GmbH) using a Hielscher Ultrasonic Processor UP400S equipped with a Sonotrode H7 probe and operating in continuous at full amplitude for 35 hours. An ice bath was used in order to avoid excessive heating of the dispersion upon ultrasonication. Afterwards, 15.0 g of the EG-EMIMAc dispersion was mixed with a 4.5% w/w solution of iron acetate-EMIMAc, and left to stir overnight at 700 rpm. The iron precursor solution was obtained by dissolving 2.6 g of iron (II) acetate (Alfa Aesar) in 55.8 g of EMIMAc under stirring condition (700 rpm) for 1 hour at 80 °C. The dispersion was placed inside a borosilicate glass reaction vials and exposed to microwave irradiation using an Anton Paar Monowave 300 microwave reactor using the procedure “heat as fast as possible” until the temperature of 230 °C was reached. The temperature was held for 180 s before cooling down to 40 °C. The solid product was separated from the residual ionic liquid through vacuum filtration using Fluoropore™ PTFE filter membranes (pore size 0.22 µm, diam. 47 mm) and rinsed with ultrapure water (Milli Q). The composite was finally obtained after a final thermal annealing step at 800 °C for 3 h in Ar atmosphere (heating rate: 5 °C min^−1^). Fe_3_O_4_ or MUG were obtained applying the same procedure but using only the iron precursor solution or the graphite dispersion, respectively.

### Physicochemical characterizations

SEM micrographs were collected with a ZEISS LEO 1550VP Field Emission Scanning Electron Microscope after Pt sputtering of the samples. XRD patterns were recorded by a Bruker D8 Advance diffractometer equipped with a CuKα source (λ = 0.154 nm) in the 10–80 2 Theta degree range measuring with a focusing Goebel mirror. TEM characterizations were carried out using an aberration (image) corrected FEI Titan 80–300 operated at 80 kV and 300 kV equipped with a Gatan imaging filter (Tridiem 863). For (S)TEM measurements, samples were prepared by dispersing a small amount of powder directly onto holey carbon Au grids (Quantifoil GmbH). EDX-EELS Elemental mapping was performed in scanning transmission electron microscopy (STEM) mode with drift correction. For the composition maps, Hartree-Slater cross-section model (283.9–337.9 eV for C K-edge and 521.5–561.4 eV for O K-edge) was used for signal quantification of C K-edge and O K-edge, whereas Hydrogenic w/ WL model (708.1–748.0 eV for Fe L_2,3_) has been employed for Fe. A Power law model has been used for the correction of background for all the three elements (224.5–279.7 eV for C K-edge, 446.2–516.4 eV for O K-edge and 631.9–694.0 eV for Fe L_2,3_). TGA analyses were conducted at scan rate of 5 °C min^−1^ under O_2_ atmosphere using a TA Instruments Q 5000. Specific surface area and pore size distribution were measured by nitrogen adsorption and calculated according to the BET and BJH theories (Autosorb IQ, Quantachrome Instruments). CHNS analyses were performed using an elementar vario MICRO cube micro-analyzer. ICP measurements were carried out using an ULTIMA 2 ICP-OES spectrometer.

### Electrodes preparation

The working electrodes were prepared by casting water-based slurries consisting of 80 wt.% active material, 10 wt.% sodium carboxymethyl cellulose (Walocel CRT 2000 PA, Dow Wolff Cellulosics) and 10 wt.% conductive carbon (Super C65, from Imerys Graphite and Carbon). After homogenizing the dispersion in an agate mortar for 15 minutes, the obtained slurry was casted on dendritic copper foil (Schlenk, 99.9%) with a wet film thickness of 120 μm. The electrode layer was dried overnight at 80 °C and subsequently punched to disc electrodes (ø = 12 mm). Finally, the electrodes were further dried in a glass oven under vacuum at 180 °C for at least 8 h. The electrodes average active material mass loading was 1 mg cm^−2^.

### Electrochemical characterizations

Electrochemical tests were carried out using 3-electrodes Swagelok T-cell assembled in an Ar filled glovebox (MBraun) with O_2_ and H_2_O levels < 0.1 ppm. Lithium metal foil (from Rockwood Lithium) was used as counter and reference electrode. Glass fibre filter separator (WhatmanGf/D) drenched with 1 M LiPF_6_ in EC:DMC 1:1 w/w electrolytic solution was employed as electrolyte (120 μL for each assembled cell). The electrochemical tests, galvanostatic charge/discharge cycles, were performed in two different potential ranges (from 0.005 V to 3 V vs. Li/Li^+^ and from 0.005 V to 1 V vs. Li/Li^+^) using a Maccor Battery Tester 4300 at 20 ± 1 °C. Different specific currents, ranging from 0.1 A g^−1^ to 50 A g^−1^ were applied. Cyclic voltammetry tests were carried out with a scan rate of 5 mV s^−1^ in a 0.005 V–3 V potential range using a VMP3 galvanostat/potentiostat by Bio-Logic (France). The same instrumentation was used to acquire the electrochemical impedance spectra (sinus amplitude: 5 mV; frequency range: 1 MHz-10 mHz) at different temperatures (in a MK 53 climatic chamber from Binder). Prior to collecting the impedance spectra, the electrodes were subject to a charge/discharge cycle at at 20 °C and 0.1 A g^−1^ (lithiation down to 5 mV and delithiation to 1 V vs. Li/Li^+^) to ensure full formation, followed by reduction up to 0.11 V vs. Li/Li^+^ and a 2 h rest step.

### Electrode density calculations

The electrodes density was calculated by considering the volume of the coating layer (without the current collector) and active material mass only (no conductive additive and binder considered).

### Average voltage calculations

The average lithiation and delithiation voltage was obtained by the integral of the voltage profiles (energy) divided by the specific gravimetric capacity.

## Additional Information

**How to cite this article**: Raccichini, R. *et al.* Boosting the power performance of multilayer graphene as lithium-ion battery anode via unconventional doping with in-situ formed Fe nanoparticles. *Sci. Rep.* **6**, 23585; doi: 10.1038/srep23585 (2016).

## Supplementary Material

Supplementary Information

## Figures and Tables

**Table 1:** C, H, N, S, Fe, O contents for expanded graphite (EG), multilayer graphene (MUG) and composite (MUG-Fe_3_O_4_).

	C^ (wt. %)	H^ (wt. %)	N^ (wt. %)	S^ (wt. %)	Fe* (wt. %)	O^#^(wt. %)
EG	93.19	–	–	–	–	6.81
MUG	90.98	0.30	–	–	–	8.72
MUG-Fe_3_O_4_	90.67	0.29	–	–	0.25	8.79

^CHNS Elemental analysis; *ICP Analysis; ^#^Content determined by subtraction.

**Table 2:** Densities of the electrodes based on commercial graphite (SLP30), multilayer graphene (MUG) or composite (MUG-Fe_3_O_4_) active material.

Active Material	Electrode density (g mL^−1^)
SLP30	0.478
MUG	0.210
MUG-Fe_3_O_4_	0.228

**Table 3:** Specific gravimetric and volumetric capacities and average delithiation voltages of graphite (SLP30), multilayer graphene (MUG) and composite (MUG-Fe_3_O_4_) at different current rates.

Cycle number(specific current)	Activematerial	Gravimetric capacity(mAh g^−1^)	Volumetriccapacity (Ah L^−1^)	Average delithiationvoltage (V)	
10^th^ (0.1 A g^−1^)	SLP30	336.3	160.7	0.197	
	MUG	345.7	72.6	0.173	
	MUG-Fe_3_O_4_	368.3	84.0	0.171	
50^th^ (1 A g^−1^)	SLP30	84.8	40.5	0.412	
	MUG	340.2	71.4	0.184	
	MUG-Fe_3_O_4_	346.3	79.0	0.189	
250^th^ (5 A g^−1^)	SLP30	20.0	9.6	0.697	
	MUG	160.3	33.7	0.303	
	MUG-Fe_3_O_4_	257.8	58.8	0.244	
