# Supplementary material for: Boosting the power performance of multilayer graphene as lithium-ion battery anode via unconventional doping with in-situ formed Fe nanoparticles
Source: Sci Rep. 2016 Mar 30;6:23585. doi: 10.1038/srep23585 (PMC4812302; doi:10.1038/srep23585)
Supplement: Supplementary Information [file srep23585-s1.pdf]

Supplementary Information

Boosting the power performance of multilayer graphene as lithium-ion battery anode via unconventional doping with in-situ formed Fe nanoparticles

Rinaldo Raccichini[a,b,c], Alberto Varzi[a,b], Venkata Sai Kiran Chakravadhanula[a,d,e], Christian Kübel[a,d,e], Stefano Passerini[a,b,*]

[a]*Helmholtz Institute Ulm (HIU), Helmholtzstrasse 11, 89081 Ulm, Germany*

[b]*Karlsruhe Institute of Technology (KIT), P.O. Box 3640, 76021 Karlsruhe, Germany*

[c]*Institute of Physical Chemistry, University of Muenster, Corrensstrasse 28/30, 48149 Muenster, Germany*

[d]*Institute of Nanotechnology (INT), Karlsruhe Institute of Technology (KIT), Hermann-von-Helmholtz Platz 1, 76344 Eggenstein-Leopoldshafen, Germany*

[e]*Karlsruhe Nano Micro Facility (KNMF), Karlsruhe Institute of Technology (KIT), Hermann-von-Helmholtz Platz 1, 76344 Eggenstein-Leopoldshafen, Germany*

Corresponding Author: Tel: +49 (0) 731 5034101, email: stefano.passerini@kit.edu

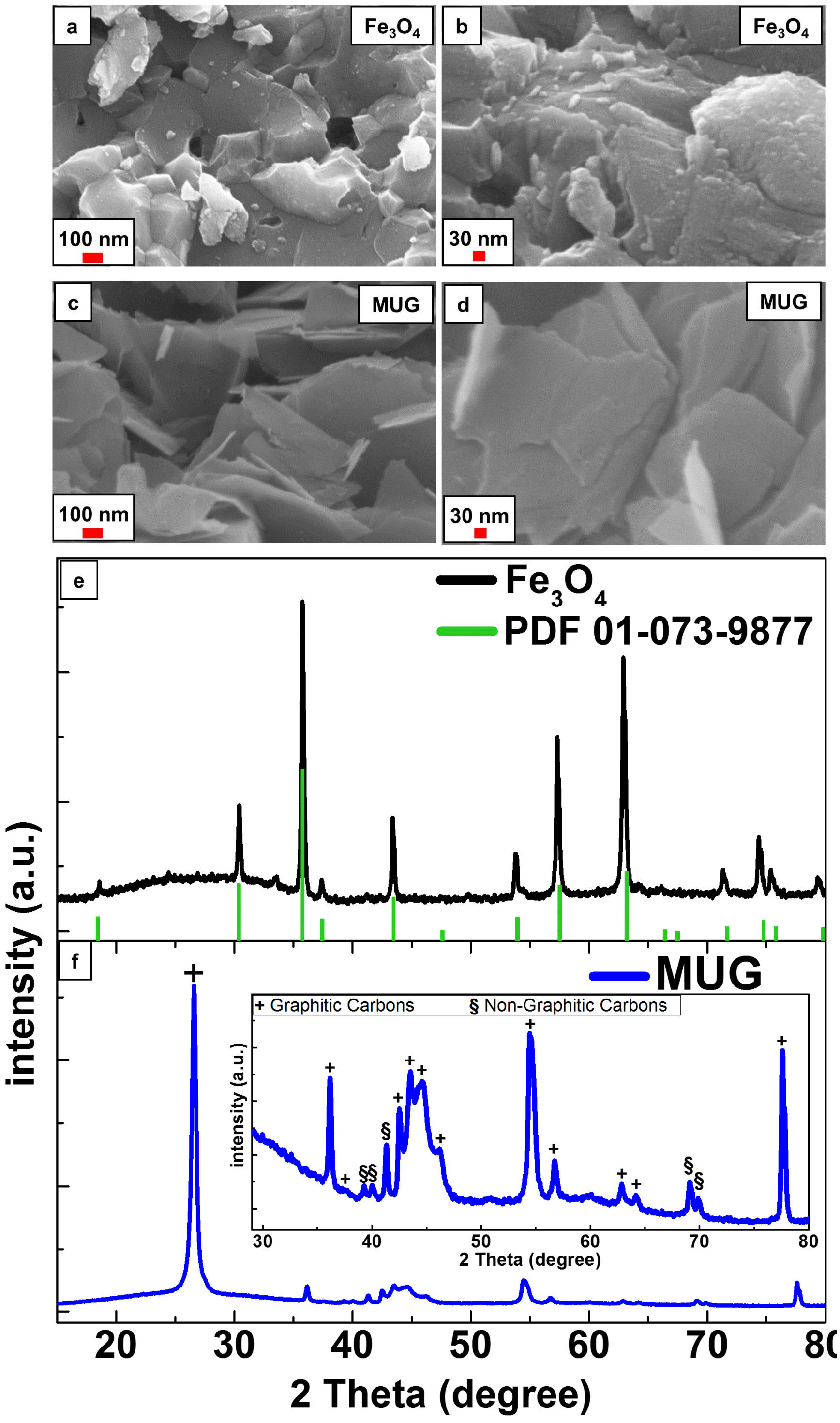

Supplementary Figure 1 | Morphological and structural characterization of Fe_3O_4 and uncarved multilayer graphene (MUG). Low- and high- magnification SEM micrographs of Fe_3O_4 (**a** and **b**) and uncarved multilayer graphene MUG (**c** and **d**). (**e**) XRD pattern of the

Fe_3O_4 and the corresponding Fe_3O_4 standard XRD data (PDF# 01-073-9877). (**f**) XRD pattern of uncarved multilayer graphene. Positions of main reflections are marked as (+) graphitic carbons; (§) non-graphitic carbons.

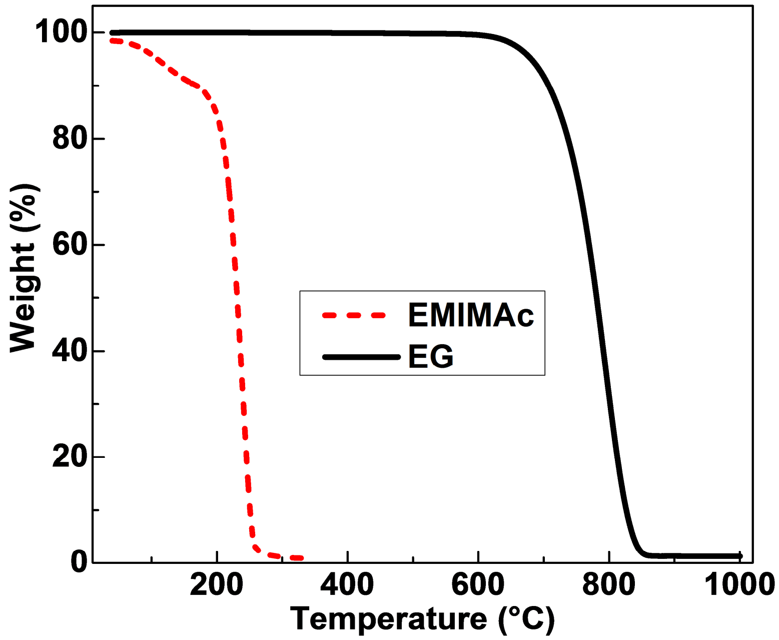

Supplementary Figure 2 | Thermal stability of ionic liquid and expanded graphite. TGA profiles for 1-ethyl-3-methylimidazolium acetate (EMIMAc) and expanded graphite (EG) at scan rate of 5 °C min^{-1} under O_2 atmosphere using a TA Instruments Q 5000.

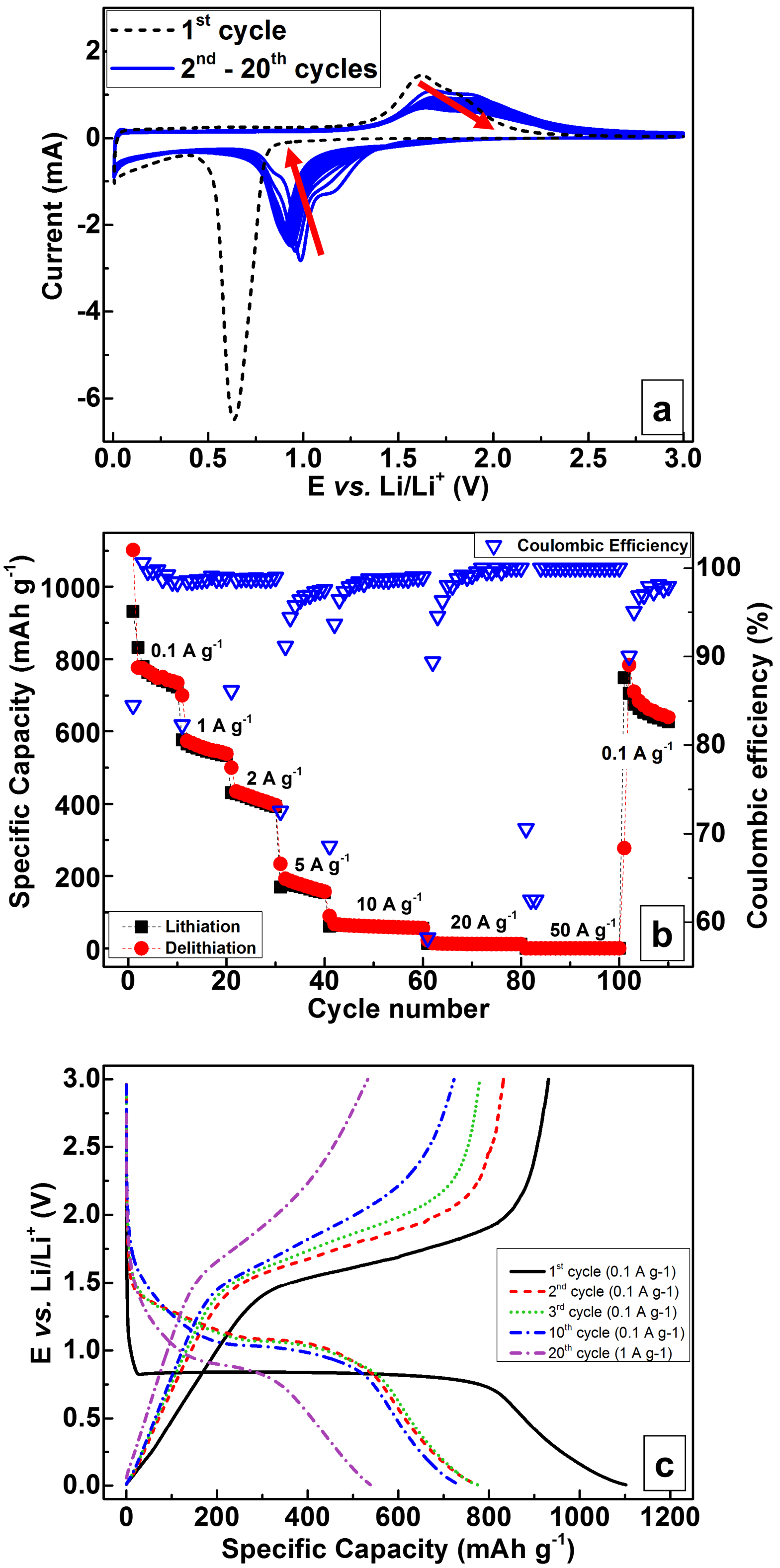

Supplementary Figure 3 | Li$^+$ storage properties of Fe_3O_4 as anode active material. (a) Cyclic voltammetries recorded by sweeping the electrode potential at a rate of 5 mV s^{-1}. The

voltammetric waves shows the typical behaviour of a conversion material: a cathodic peak in the 1st cycle, at ca. 0.63 V, related to a first partially reversible conversion reaction[1,2], which could be written as:

$$Fe_3O_4 \xrightarrow{Li^+} LiFe_3O_4 \xrightarrow[(0 < x \leq 7)]{xLi^+} Li_{1+x}Fe_{3-3x/7}O_4 \longleftrightarrow 4Li_2O + 3Fe^0$$

The corresponding reverse reaction is detected as a broad wave between 1.4 V and 2.1 V. Here, lithium oxide and metallic iron are converted to the intermediate Li-Fe-O phase ($Li_{1+x}Fe_{3-3x/7}O_4$), only. The initial Fe_3O_4 phase is never formed again[1,3]. The peaksô evolution in the following cycles testifies the already known structural re-organization of the nanomaterial[4]. (**b**) Specific gravimetric capacities obtained upon various galvanostatic lithiation/delithiation cycles performed at different current rates. Fe_3O_4 shows high values of about 1100 mAh g^{-1} and 930 mAh g^{-1} for the 1st lithiation and delithiation cycles, respectively, at the specific current of 0.1 A g^{-1}. However, it suffers from considerable capacity fading and low Coulombic efficiency. Moreover, it displays a rather poor rate capability (only 20% capacity retention at 5 A g^{-1}). (**c**) Potential profiles of selected cycles highlight the large voltage hysteresis between lithiation and delithiation, typical of conversion materials. This accounts for about 0.8 V and 1.4 V in the 1st and 20th cycle, respectively. All measurements were performed in Li half-cell with a 1 M $LiPF_6$ - EC:DMC 1:1 w/w electrolyte in the potential range comprised between 0.005 V and 3 V.

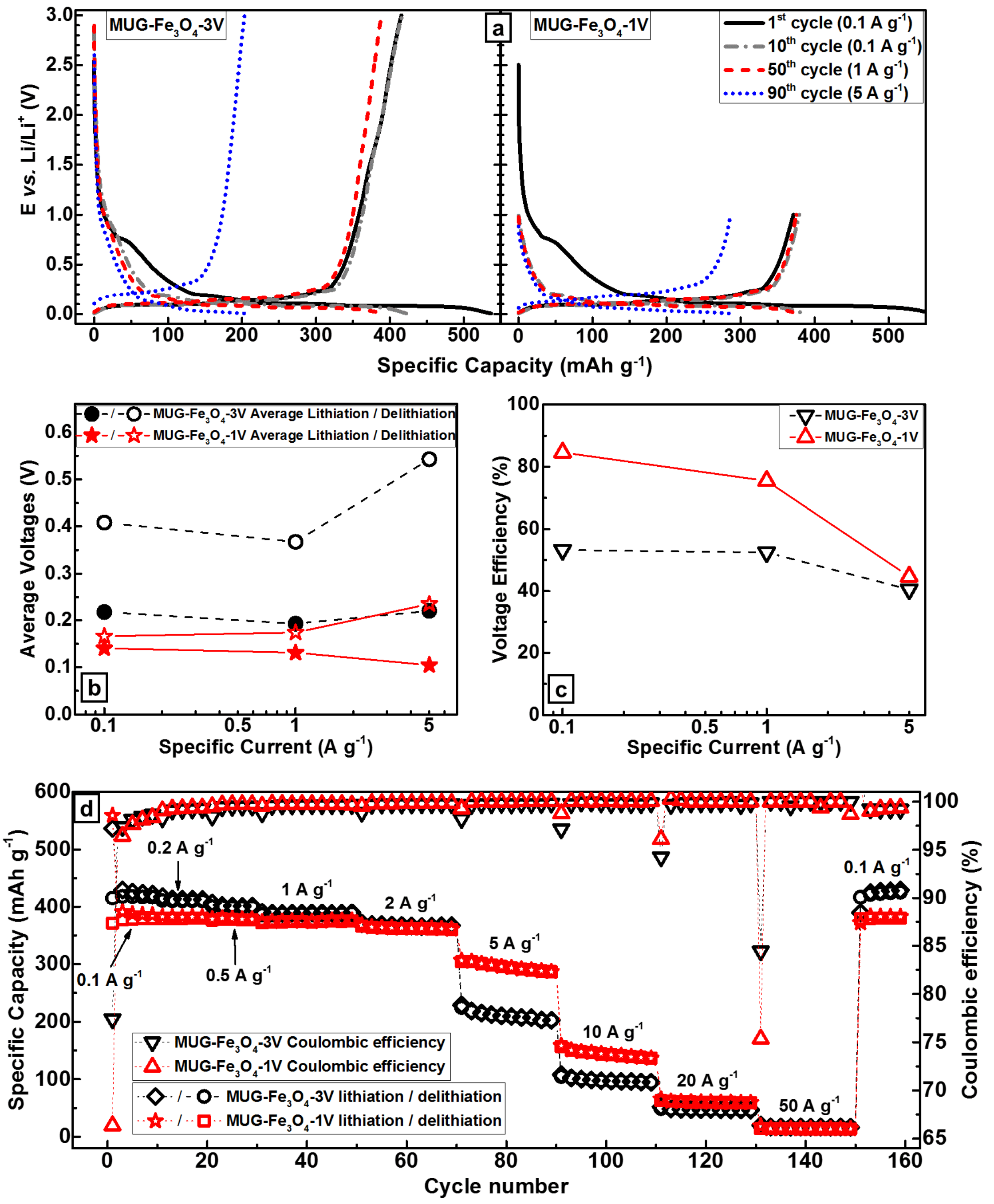

Supplementary Figure 4 | MUG-Fe_3O_4 composite rate capability tests at different cut-off potentials. (**a**) Potential profiles of selected cycles in two different potential ranges: 0.005 Vï 3 V (left) and 0.005 Vï 1 V (right) at different specific currents. Upon the 1st lithiation, a short plateau at about 0.75 V indicates the Fe_3O_4 conversion reaction occurring in both electrodes. Upon the following delithiation, a minor feature at about 1.5 V, related to the

conversion process, is observed only for the electrode charged up to 3 V. Obviously, the electrode charged to 1 V does not show such a feature, and the iron generated during the 1st lithiation is retained in metallic form. (**b**) Average lithiation voltages (ALVs) and average delithiation voltages (ADVs) for the selected cycles in **b**. For the electrode charged up to 1 V, at specific currents Ò1 A g^{-1}, the ADV is always below 0.180 V while, at 5 A g^{-1}, an increase up to 0.235 V could be noticed. Similarly, the ADV never exceeds 0.141 V. Conversely, the electrode charged to 3 V shows ADV and ALV values in the ranges of 0.408-0.543 V and 0.217-0.220 V, respectively. (**c**) Voltage efficiencies (VEs) for the selected cycles in **b**. The electrode charged to 1 V shows a slight decrease of VE upon cycling ranging from 84.6 % and 44.7 % for the specific current increase from 0.1 A g^{-1} to 5 A g^{-1}, respectively. On the contrary, the electrode charged to 3 V has a constant VE. However, its maximum voltage efficiency is only of 53.2 % (at specific current of 0.1 A g^{-1}). Although the VE at the highest current (i.e., 5 A g^{-1}) are for both materials quite similar, the average lithiation/delithiation voltages of the electrode cycled with the 1 V cut-off are much lower. (**d**) Rate capability obtained with the two different cut-off voltages. As expected, during the 1st lithiation, both electrodes show a similar specific capacity of about 550 mAh g^{-1}. In the following delithiation step, the electrode charged up to 3 V showed higher coulombic efficiency (77.4% instead of 66.4%) associated to the reconversion of Fe^0 to the Li-Fe-O phase. After the initial 10 cycles (0.1 A g^{-1}) the Coulombic efficiency of both electrodes stabilizes at 99 % with delivered capacity of 416.7 mAh g^{-1} and 379.3 mAh g^{-1} for the wider and narrower potential ranges, respectively. When the current rate is increased up to 2.0 A g^{-1}, the electrode charged up to 3V shows higher capacities resulting from the conversion material. For higher loads (i.e., 5 A g^{-1} and 10 A g^{-1}), however, the electrode charged at 1 V outperforms the one charged up to 3 V. Finally, after the rate test, both electrodes seem to fully recover their capacity.

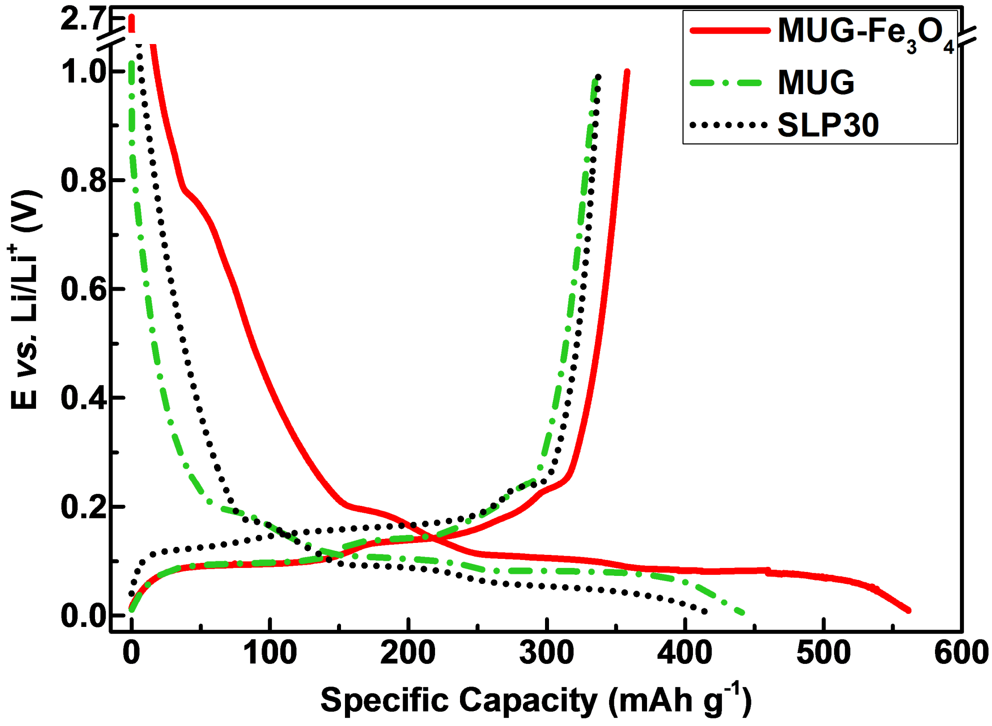

Supplementary Figure 5 | 1st cycle voltage profiles for commercial graphite (SLP30), multilayer graphene (MUG) and composite (MUG-Fe_3O_4) in the 0.005 V–1 V potential window for an applied specific current of 0.1 A g^{-1}. Upon the 1st lithiation, MUG-Fe_3O_4 shows a short plateau at about 0.75 V indicating the conversion reaction of Fe_3O_4. Between 0.2 V and 0.005 V a sequence of plateaus indicate the different lithium intercalation stages (see Supplementary Table 1) in the multilayer structure of graphene. MUG and SLP30 also show a similar sequence of plateaus below the 0.2 V potential while, because of the iron oxide absence, no plateaus are detected above 0.2 V for both materials. Upon the following delithiation, MUG and MUG-Fe_3O_4 shown similar low-polarization profiles up to 0.15 V. Differently, SLP30 displays a slightly higher polarization. However, the different lithium intercalation stages (similar to the ones of MUG and MUG-Fe_3O_4) can still be distinguished.

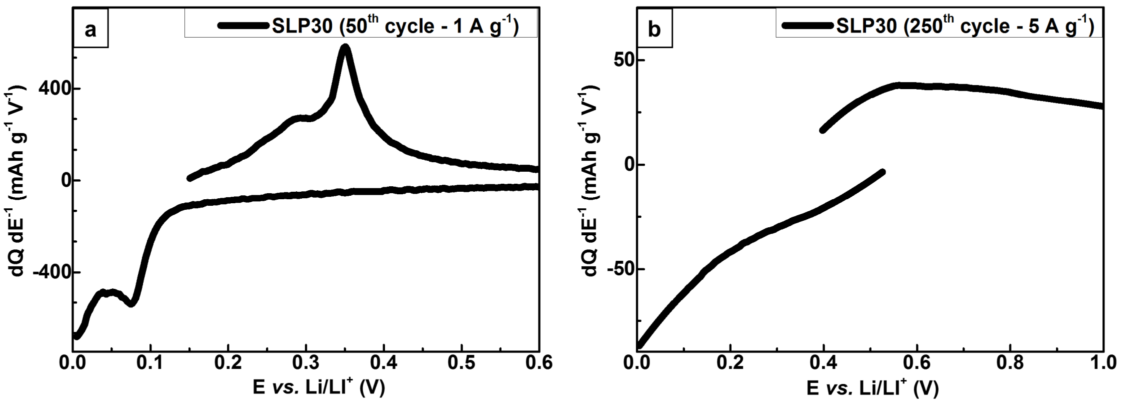

Supplementary Figure 6 | Differential capacity plots of commercial graphite SLP30. (**a**) Calculated dQ dE^{-1} vs E differential profiles for the 50th cycle of graphite (SLP30) in the potential range 0.005 V - 0.6 V. (**b**) Calculated dQ dE^{-1} vs E differential profiles for the 250th cycle of graphite (SLP30) in the potential range 0.005 V-1 V.

Li^{+} storage mechanism	Intercalation stage	Lithiation voltage region	Delithiation voltage region
$3LiC_{72} + 3Li^{+}$ ź $6LiC_{36}$	VIII ź IV	0.300-0.130	0.165-0.300
$6LiC_{36} + 2Li^{+}$ ź $8LiC_{27}$	IV ź IIIa		
$8LiC_{27} + 4Li^{+}$ ź $12LiC_{18}$	IIIa ź IIIb	0.130-0.090	0.125-0.165
$12LiC_{18} + 6Li^{+}$ ź $18LiC_{12}$	IIIb ź II		
$18LiC_{12} + 18Li^{+}$ ź $36LiC_{6}$	II ź I	0.090-0.005	0.005-0.125

Supplementary Table 1 | Typical "graphite-like" lithium intercalation staging observed in the MUG-Fe_3O_4 composite.

Supplementary References

1. Thackeray, M. M. *et al.* Intermetallic Negative Electrodes for Lithium Batteries. *Electrochem. Soc. Proceeding* **36,** 92ï 101 (2000).

2. Zhang, M. & Jia, M. High rate capability and long cycle stability Fe_3O_4ï graphene nanocomposite as anode material for lithium ion batteries. *J. Alloys Compd.* **551,** 53ï 60 (2013).

3. Thackeray, M. M., David, W. I. F. & Goodenough, J. B. Structural characterization of the lithiated iron oxides $Li_xFe_3O_4$ and $Li_xFe_2O_3$ $(0<x<2)$. *Mater. Res. Bull.* **17,** 785ï 793 (1982).

4. Cabana, J., Monconduit, L., Larcher, D. & Palacín, M. R. Beyond intercalation-based Li-ion batteries: The state of the art and challenges of electrode materials reacting through conversion reactions. *Adv. Mater.* **22,** 170ï 192 (2010).
